# Comprehensive analysis of current leakage at individual screw and mixed threading dislocations in freestanding GaN substrates

**DOI:** 10.1038/s41598-023-29458-3

**Published:** 2023-02-10

**Authors:** Takeaki Hamachi, Tetsuya Tohei, Yusuke Hayashi, Masayuki Imanishi, Shigeyoshi Usami, Yusuke Mori, Akira Sakai

**Affiliations:** 1 Graduate School of Engineering Science, Osaka University, 1-3 Machikaneyama-Cho, Toyonaka, Osaka 560-8531 Japan; 2 Graduate School of Engineering, Osaka University, 2-1 Yamadaoka, Suita, Osaka 565-0871 Japan

**Keywords:** Materials for devices, Electronic devices

## Abstract

The electrical characteristics of Schottky contacts on individual threading dislocations (TDs) with a screw-component in GaN substrates and the structures of these TDs were investigated to assess the effects of such defects on reverse leakage currents. Micrometer-scale platinum/GaN Schottky contacts were selectively fabricated on screw- and mixed-TD-related etch pits classified based on the pit size. Current–voltage (*I–V*) data acquired using conductive atomic force microscopy showed that very few of the screw TDs generated anomalously large reverse leakage currents. An analysis of the temperature dependence of the *I–V* characteristics established that the leakage current conduction mechanisms for the leaky screw TDs differed from those for the other screw and mixed TDs. Specifically, anomalous current leakage was generated by Poole–Frenkel emission and trap-assisted tunneling via distinctive trap states together with Fowler–Nordheim tunneling, with the mechanism changing according to variations in temperature and applied voltage. The leaky TDs were identified as Burgers vector **b** = 1***c*** closed-core screw TDs having a helical morphology similar to that of other screw TDs generating small leakage currents. Based on the results, we proposed that the atomic-scale modification of the dislocation core structure related to interactions with point defects via dislocation climbing caused different leakage characteristics of the TDs.

## Introduction

Gallium nitride (GaN) is one of the most promising materials for the fabrication of high-power electronic devices as a consequence of the superior physical properties of this material, such as a wide bandgap, high breakdown electric field and good electron mobility^1,2^. However, due to the significant mismatches between the lattice constants and thermal expansion coefficients of GaN crystals and various other compounds, it has been difficult to grow high-quality bulk GaN substrates. In particular, the presence of threading dislocations (TDs) in GaN crystals often degrades the performance of GaN-based devices^3–7^. Many studies have found that TDs with a screw-component (i.e. ***c***-component Burgers vectors **b**) generally cause current leakage in GaN crystals^3–5,8–12^. Even so, it should be noted that the types of TDs observed to induce leakage currents have differed between various studies. In early studies using conductive atomic force microscopy (C-AFM), screw and mixed TDs and/or nanopipes (i.e. open-core screw TDs) behaved as leakage current paths in GaN thin films with high TD densities of 10^8-9^ cm^-2^^8–10^. Screw and mixed TDs also caused initial leakage current in vertical GaN-on-GaN Schottky barrier diodes^4^. In the case of vertical GaN-on-GaN pn diodes with lower TD densities of 10^6-7^ cm^-2^, Usami *et al*. reported that pure screw TDs, particularly having **b**=1***c***, served as leakage paths^5^. In contrast, the same group found that, in other pn diodes, the majority of the leakage spots coincided with nanopipes while other types of TDs, including pure screw TDs, did not cause a current leakage^11,12^. In the latter study, it was also established that nanopipes that did not generate leakage current were present in the same device and that the correlation between leakage spots and nanopipes was dependent on the GaN growth conditions^11^. The inconsistent leakage behavior of TDs observed in these studies strongly suggests that the effects of the TDs on GaN-based power devices are sensitive to the growth conditions^13^ and thereby not solely determined by the type of TD. Specifically, factors such as the Burgers vector^5^, core structure^14^, inclination direction^15,16^ and strain field of the dislocations^17^, as well as point defects interacting with the TDs^4,10,17–19^, may influence the electrical characteristics of these defects. Unfortunately, few studies have examined the detailed relationships between such structural characteristics and the electrical properties of TDs, primarily because of challenges related to quantitative electrical measurements of individual TDs. Recently, we developed a technique that enables assessments of the electrical properties of micrometer-scale Schottky contacts locally formed on individual TDs. This process is based on a combination of an etch pit method, a local metal deposition technique using a focused ion beam (FIB) system, and C-AFM^17,18^. In addition, our group recently identified a one-to-one correspondence between the etch pit size and the Burgers vector of TDs in GaN bulk crystals grown by hydride vapor phase epitaxy (HVPE)^20^. The results of these prior studies allow the selective formation and electrical assessment of Schottky contacts on individual screw and mixed TDs having a ***c***-component in the Burgers vector (i.e., **b**=1***c***, 1***a***+1***c*** and 1***m***+1***c***). The present study evaluated electrical characteristics of Schottky contacts and detailed structures of screw and mixed TDs in a GaN substrate to elucidate the effects of such defects on reverse leakage currents.

## Methods

**Figure 1:**
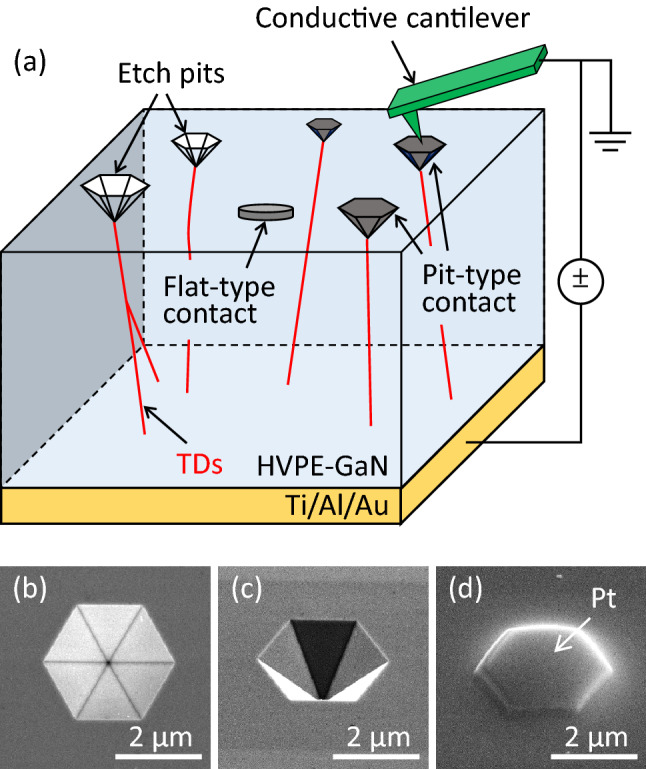
(**a**) Schematic of the C-AFM measurement setup used to evaluate the electrical properties of local Schottky contacts. Pit- and flat-type contacts were formed at etch pits related to TDs and at surface sites where no etch pits were present, respectively. Scanning electron microscopy images showing (**b**) a plan-view and (**c**), (**d**) bird’s-eye views of a typical etch pit (**b**), (**c**) before and (d) after the deposition of Pt.

Figure 1a shows the experimental process used to examine the electrical properties of local Schottky contacts, employing a Si-doped n-type GaN bulk crystal similar to samples previously evaluated by our group^20^. This crystal was grown homoepitaxially via HVPE on a Na-flux GaN substrate produced using a combination of flux-film-coating and multipoint-seed-coalescence techniques^21^. The *c*-plane and back surfaces of the specimen were subjected to chemical–mechanical and mechanical polishing, respectively, to obtain a freestanding GaN bulk crystal with an HVPE-grown layer having a thickness of approximately 200 µm. The carrier concentration (*N*_D_) in the HVPE-grown GaN was determined to be 1.66 $\times$ 10^18^ cm^-3^ based on capacitance–voltage (*C-V*) analyses performed at room temperature. The locations of TDs were determined based on the formation of etch pits with inverted hexagon morphologies and several micrometers in diameter at each TD by wet chemical etching with a eutectic mixture of sodium hydroxide and potassium hydroxide at 450 °C for 20 min. Images of these pits are provided in Fig. 1a–c. The etch pit density in the specimen was approximately 7 $\times$ 10^5^ cm^-2^. Prior to forming an ohmic contact, the GaN crystal was cleaned with a sulfuric peroxide mixture and with hydrofluoric acid, rinsed with deionized water and dried under a stream of gaseous nitrogen. Multilayers having the composition titanium/aluminum/gold (40/40/200 nm) were formed on the backside of the GaN substrate by sputtering deposition to make an ohmic contact. Platinum (Pt) was deposited into each etch pit using an electron-beam assisted deposition technique employing a FIB system (FEI Versa™ 3D DualBeam™) so that conformal micrometer-scale Schottky contacts were formed on the individual TDs, as shown in Fig. 1a, d. Hereafter, this type of Schottky contact is referred to as a pit-type contact. Our previous work determined that the size of such etch pits in the same type of HVPE-grown GaN crystal was correlated with the Burgers vector of the TD^20^. Specifically, **b** = 1***a***, **b** = 1***a*** + 1***c***, **b** = 1***c*** and **b** = 1*** m*** + 1***c*** were associated with extra small (XS), small (S), medium (M) and large (L) sizes, respectively. To assess the screw and mixed TDs that are often reported to act as preferential leakage paths in GaN-based power devices and crystals^3–5,8–14,17^, S-, M- and L-etch pits were selected. A total of 45, 45 and 11 S-, M- and L-pit-type contacts were analyzed in this study, with equivalent areas of 0.06, 0.23 and 0.31 mm^2^, respectively, based on the density of each type of pit. As indicated in Fig. 1a, circular Pt/GaN contacts having a diameter of approximately 5 µm were also formed on surface sites at which there were no etch pits. That is, these contacts did not involve a TD at the Schottky interface. Hereafter, this type of Schottky contact is referred to as a flat-type contact. After annealing the sample at 200 °C for 2 min in air to sinter the as-deposited Pt electrodes, the local current–voltage (*I-V*) characteristics were evaluated using C-AFM (Hitachi AFM5300E) with a conductive rhodium-coated cantilever under vacuum at room temperature (see Fig. 1a). In addition, the temperature dependence of *I-V* characteristics (*I-V-T*) of representative Schottky contacts were ascertained. The electric field distributions around each Schottky interface for the pit-type contacts were calculated employing a finite element simulation with the COMSOL Multiphysics software package.

Following the C-AFM assessments, multi-photon excitation photoluminescence microscopy (MPPL, Nikon A1MP instrument) was used to investigate the three-dimensional propagation morphologies of the TDs in the bulk crystals. These trials used a laser wavelength of 800 nm and monitored photoluminescence at wavelengths ranging from 352 to 388 nm. After these MPPL trials, several thin samples incorporating TDs were prepared for transmission electron microscopy (TEM) observations using a FIB. The microstructures and Burgers vectors of the TDs were identified based on a conventional ***g*** $\cdot$ **b** analysis, weak-beam dark-field imaging and large-angle convergent-beam electron diffraction (LACBED) techniques using TEM (JEOL JEM-2100).

## Results

### J-V characteristics of Schottky contacts at room temperature

**Figure 2:**
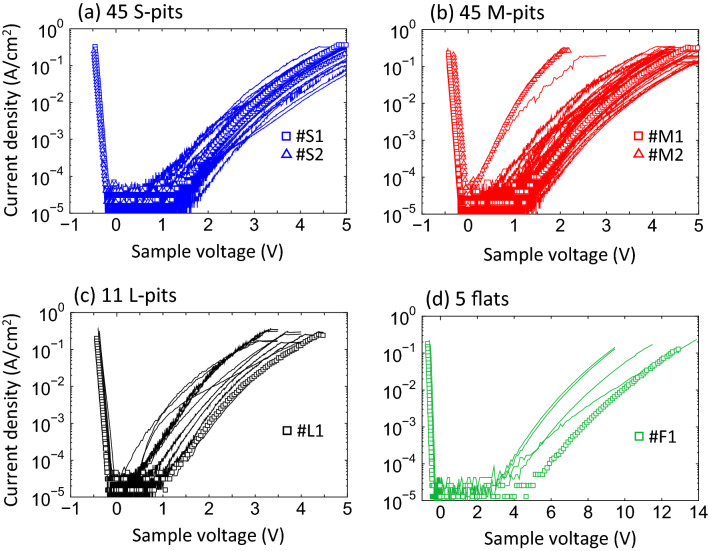
*J-V* characteristics of (**a**) 45 S-pits, (**b**) 45 M-pits, (**c**) 11 L-pit-type contacts, and (**d**) 5 flat-type contacts as determined under vacuum at 293 K. The symbol plots indicate the Schottky contacts for which *I-V-T* measurements were also performed.

Figure 2a–d show the current density–voltage (*J-V*) data obtained from the 45 S-pits, 45 M-pits, 11 L-pits and five flat-type contacts. Note that the pit-type contact area was calculated as the total area of the six pyramidal planes of the etch pit and the unintentionally spread-out deposition area of Pt in a circle around each etch pit^17^. Each of these *J-V* curves exhibits rectifying characteristics. The reverse characteristics show that two of the 45 M-pits generated anomalously large leakage currents (Fig. 2b) while none of the S- or L-pit contacts exhibited this property (Fig. 2a,c). We confirmed that the shape (such as a diameter and an apex angle), surface condition of the pits before the deposition and the Pt electrode formed on the pits for the leaky M-pits were similar to those for the less-leaky ones by scanning electron microscopy. Therefore, it is unlikely that the anomalous leakage properties for the two M-pits are local experimental errors. The density of the anomalous M-pits was estimated to be 8.9 $\times$ 10^2^ cm^-2^, corresponding to 4.5% of all M-pits and 0.14% of all etch pits. Based on our previous work, it is likely that screw TDs having **b** = 1***c*** were situated below these M-pits, while mixed TDs having **b** = 1***a*** + 1***c*** and **b** = 1*** m*** + 1***c*** were present under the S- and L-pits^20^. This result indicates that mixed TDs are less likely to be responsible for significant reverse leakage currents in GaN crystals.

### TEM analysis of the leaky M-pit

**Figure 3:**
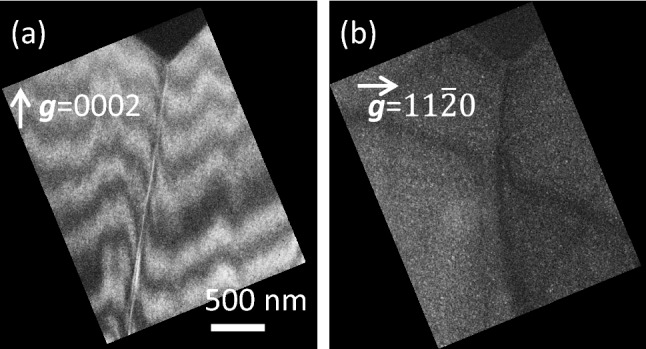
Cross-sectional weak-beam dark-field TEM images of TDs under the leaky M-pit (i.e., #M2) acquired with (**a**) ***g*** = 0002 and with (**b**) ***g*** = 11$\overline{2 }$0.

Figure 3a and b provide the results of ***g*** $\cdot$ **b** analyses and show weak-beam dark-field TEM images of the TD under the leaky M-pit (referred to as #M2 hereafter) acquired with diffraction vectors ***g*** = 0002 and ***g*** = 11$\overline{2 }$ 0, respectively. Because the dislocation line is visible in the ***g*** = 0002 image but invisible in the ***g*** = 11$\overline{2 }$ 0 image, the TD was identified as screw-type according to the invisibility criterion. Figure 3a also demonstrates that this TD had a closed-core rather than open-core structure. LACBED analysis demonstrated that the Burgers vector was **b** = 0001 = 1***c***, as shown in Supplementary Figs. S1(a) and S1(b) online*.* As a result, it was revealed that the Burgers vector and apparent core structures (in terms of open-core or closed-core) of the screw TD under the leaky M-pit were very similar to those of screw TDs under other M-pits which generated small leakage currents^20^.

### Forward J-V-T characteristics of local Schottky contacts

**Figure 4:**
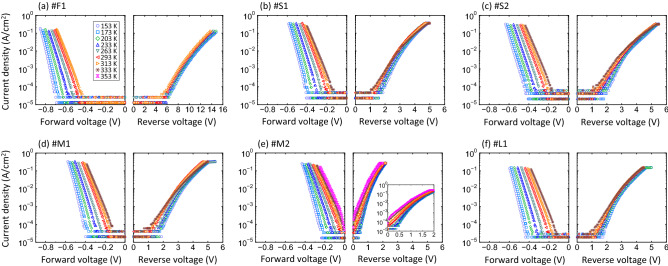
*J-V-T* characteristics of the (**a**) #F1, (**b**) #S1, (**c**) #S2, (**d**) #M1, (**e**) #M2 and (**f**) #L1 pits.

Figure 4a–f present *J-V-T* data obtained for one flat-type contact (labeled as #F1), two S-pits (#S1 and #S2), two M-pits (#M1 and #M2) and one L-pit (#L1). The room-temperature *J-V* data for these contacts are indicated by the unfilled symbols in Fig. 2a–d. The #M2 site was a pit-type contact showing a significant leakage current while the other four locations (#S1, #S2, #M1 and #L1) were representative pit-type contacts that were associated with small leakage currents. All the *J-V-T* curves showed temperature-dependent characteristics under both forward and reverse bias conditions. It should be noted that, although the measurement temperature was varied from 153 to 373 K for all contacts, some of these sites degraded irreversibly with regard to both forward and reverse characteristics, especially after high-temperature measurements over 333 K. The *J-V-T* data shown in Fig. 4a–f were obtained within the temperature range over which the *I-V* characteristics were found to be highly reproducible.

**Figure 5:**
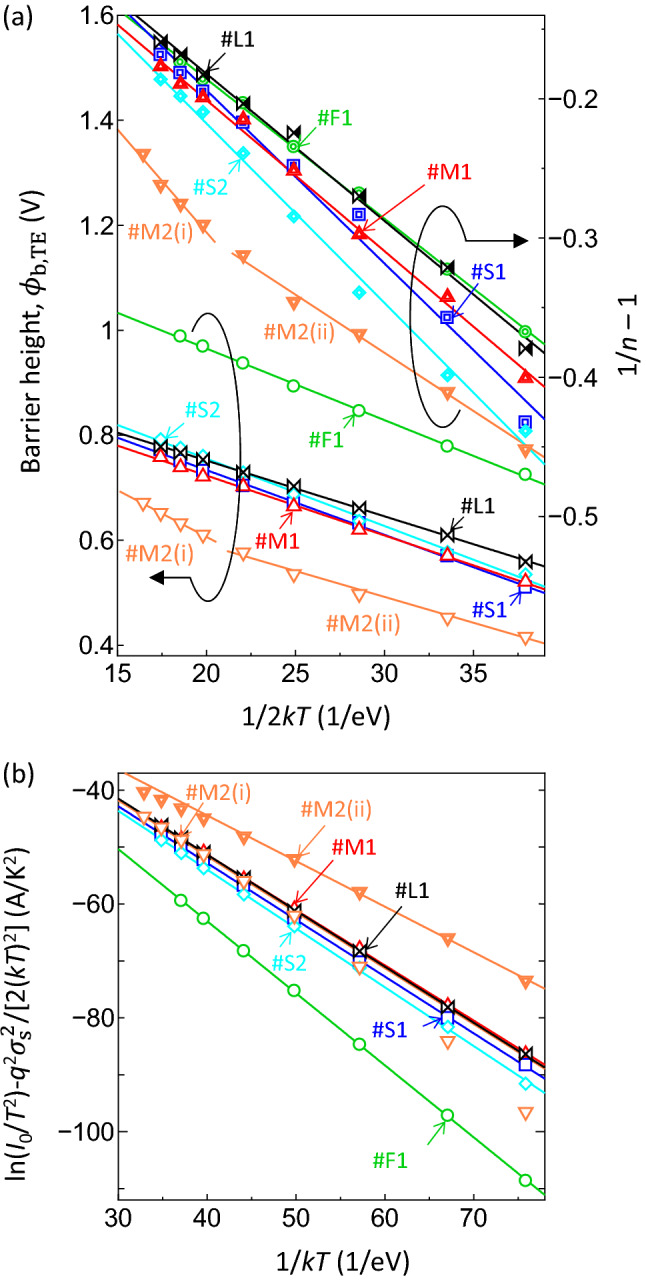
(**a**) ${\phi }_{\mathrm{b},\mathrm{ TE}}$ and $1/n-1$ vs. $1/2kT$ plots and (**b**) modified Richardson plots based on the barrier inhomogeneity model and the forward *J-V-T* characteristics for the #F1, #S1, #S2, #M1, #M2 and #L1 pits. Solid lines indicate the best fit to each plot.

In the case of ideal Schottky contacts, the values of the barrier height (${\phi }_{\mathrm{b},\mathrm{ TE}}$), ideality factor (*n*), and effective Richardson constant (${A}^{*}$) were derived from the forward *J-V-T* dataset in Fig. 4a–f based on a conventional thermionic emission (TE) model and a conventional Richardson plot. (see Supplementary Eqs. (S2)-(S4))^22^. However, the Richardson plots generated using the present experimental data were nonlinear in the lower temperature region and the calculated effective Richardson constant values were one or two orders of magnitude larger than the theoretical one. This type of non-ideal characteristics is often explained by the spatial inhomogeneity of the Schottky barrier height values (see Supplementary Eqs. (S5) and (S6))^23–25^. In this model, the barrier height at the Schottky interface is assumed to have a Gaussian distribution with the zero-bias mean barrier height (${\overline{\phi }}_{\mathrm{b}0}$) and zero-bias standard deviation of the barrier height distribution (${\sigma }_{\mathrm{s}}$). As shown in Fig. 5a, the plots of ${\phi }_{\mathrm{b},\mathrm{ TE}}$ vs. $1/2kT$ and $1/n-1$ vs. $1/2kT$ for #F1, #S1, #S2, #M1 and #L1 exhibit good linearity over the entire temperature range, indicating a single Gaussian distribution of the barrier heights. The values of voltage coefficient for the mean barrier height, ${\rho }_{2}$, and for the standard deviation, ${\rho }_{3}$, obtained from linear fits to the $1/n-1$ vs. $1/2kT$ plots range from 0.100 to 0.114 and from 0.015 to 0.055 V, respectively, and so are close to one another (with the exception of the values for pit #M2). The values of ${\overline{\phi }}_{\mathrm{b}0}$ and ${\sigma }_{\mathrm{s}}$ determined from the linear fits to plots of ${\phi }_{\mathrm{b},\mathrm{ TE}}$ vs. $1/2kT$ for the #S1, #S2, #M1 and #L1 pits are in the range from 0.95 to 1.01 V and from 0.103 to 0.113 V, respectively, while those for #F1 are 1.24 V and 0.117 V, respectively. As can be seen from Fig. 5b, the Richardson plots modified based on the inhomogeneous barrier height model (see Supplementary Eq. (S7)) for #F1, #S1, #S2, #M1 and #L1 using ${\sigma }_{\mathrm{s}}$ are also highly linear throughout the entire temperature range. The ${A}^{*}$ values calculated from the fits to the modified Richardson plots range from 8.98 to 11.06 A/cm^2^ $\cdot$ K^2^. The ${\overline{\phi }}_{\mathrm{b}0}$ obtained from the modified Richardson plots (ranging from 0.97 to 1.27 V) are almost the same as those calculated on the basis of the ${\phi }_{\mathrm{b},\mathrm{ TE}}$ vs. $1/2kT$ plots, while the ${\overline{\phi }}_{\mathrm{b}0}$ value for pit #F1 is higher than those ascertained for pit-type contacts. The calculated values of ${\rho }_{2}$, ${\rho }_{3}$, ${\overline{\phi }}_{\mathrm{b}0}$, ${\sigma }_{\mathrm{s}}$ and ${A}^{*}$ are summarized in Table 1.

**Table 1:** The ${\rho }_{2}$, ${\rho }_{3}$, ${\overline{\phi }}_{\mathrm{b}0}$, ${\sigma }_{\mathrm{s}}$ and ${A}^{*}$ values calculated from linear fits to the experimental plots of ${\phi }_{\mathrm{b},\mathrm{ TE}}$ vs. $1/2kT$ and $1/n$-1 vs. $1/2kT$ and the modified Richardson plots for all contacts.

Label	${\rho }_{2}$	${\rho }_{3}$(V)	${\sigma }_{\mathrm{s}}$(V)	${\overline{\phi }}_{\mathrm{b}0}$(V) (${\phi }_{\mathrm{b},\mathrm{TE}}$ vs. 1/2*kT*)	${\overline{\phi }}_{\mathrm{b}0}$(V) (Richardson plot)	${A}^{*}$(A/K^2^ cm^2^)
#F1	0.100	0.015	0.117	1.24	1.27	8.98
#S1	0.112	0.055	0.111	0.98	1.00	9.45
#S2	0.114	0.040	0.113	1.01	1.03	11.06
#M1	0.104	0.016	0.107	0.95	0.97	11.02
#M2(i)	0.121	-0.001	0.134	0.97	0.98	12.84
#M2(ii)	0.092	-0.128	0.099	0.79	0.80	11.94
#L1	0.103	0.029	0.103	0.96	0.98	10.09

As shown for the #M2 pit in Fig. 5a, two linear equations with different slopes could be fit to the $1/n-1$ vs. $1/2kT$ plot and the ${\phi }_{\mathrm{b},\mathrm{ TE}}$ vs. $1/2kT$ plot within the temperature ranges of above 293 K (range (i)) and below 263 K (range (ii)). These fits yielded two sets of ${\rho }_{2}$ and ${\rho }_{3}$ and ${\overline{\phi }}_{\mathrm{b}0}$ and ${\sigma }_{\mathrm{s}}$, respectively. On the basis of the $1/n-1$ vs. $1/2kT$ plot, ${\rho }_{2}$ and ${\rho }_{3}$ were calculated to be 0.121 and -0.001 V in range (i) and 0.092 and -0.128 V in range (ii), respectively. From the ${\phi }_{\mathrm{b},\mathrm{ TE}}$ vs. $1/2kT$ plot, ${\overline{\phi }}_{\mathrm{b}0}$ and ${\sigma }_{\mathrm{s}}$ were determined to be 0.97 and 0.134 V in range (i) and 0.79 and 0.099 V in range (ii), respectively. The respective modified Richardson plots are presented in Fig. 5b. The best linear fits to each plot yielded ${A}^{*}$ and ${\overline{\phi }}_{\mathrm{b}0}$ of 12.84 A/cm^2^ $\cdot$ K^2^ and 0.98 V for range (i) and 11.94 A/cm^2^ $\cdot$ K^2^ and 0.80 V for range (ii), respectively. The values for pit #M2 are also summarized in Table 1.

The ${A}^{*}$ values calculated from the modified Richardson plots for each contact (including #M2(i) and #M2(ii)) were close to the theoretical value of 24.0 A/K^2^cm^2^, while those obtained from the conventional Richardson plot were very different from the expected value. Therefore, spatially inhomogeneous barrier heights were formed at all the interfaces and had a primary effect of determining electron conduction under the forward bias condition. The barrier heights for location #F1 and the pit-type contacts showing a small leakage current (#S1, #S2, #M1 and #L1) were found to have single Gaussian distributions. Because the ${\overline{\phi }}_{\mathrm{b}0}$ values (0.95–1.03 V) and ${\sigma }_{\mathrm{s}}$ (0.103–0.117 V) for the #S1, #S2, #M1 and #L1 pits in particular were relatively similar, these forward characteristics likely resulted primarily from the TE conduction of electrons in conjunction with similar Gaussian barrier height distributions. This result strongly suggests that, in the case of the majority of the screw and mixed TDs, variations in the Burgers vector did not greatly affect the barrier height distribution or the forward electrical transport mechanism across the Schottky contacts. In contrast, the ${\overline{\phi }}_{\mathrm{b}0}$ value for pit #M2 within the high temperature range above 293 K (range (i)) was comparable to those for the other pit-type contacts but was approximately 0.18 V lower than those for the other pit-type contacts in the low temperature range below 263 K (range (ii)). These findings are in agreement with previous studies of GaN-based Schottky contacts, which found temperature-dependent Gaussian distributions that were attributed to a bimodal Gaussian barrier height distributions at Schottky interfaces^23,24^. The characteristics observed for pit #M2 in the present work could result from a change in the dominant forward electrical conduction mechanism with variations in temperature. That is, at high temperatures above 293 K corresponding to region (i), the TE process associated with thermally excited electrons surmounting the barrier height is primarily responsible for electrical conduction. However, other mechanisms such as trap-assisted tunneling (TAT) are primarily responsible for electron transport at low temperatures (below 263 K, equivalent to region (ii)). This effect, in turn, causes the apparent reduction in the barrier height^24^. As discussed later in the reverse characteristics, specific trap states which are significantly different from those for the other pit-type contacts are found to be associated with the large leakage current conduction for pit #M2.

Prior reports have ascribed inhomogeneity of the barrier height to factors such as surface defects, surface treatments (cleaning, etching, etc.), the specific metal employed and the deposition process (evaporation, sputtering, etc.). These factors influence the interface composition/phase, interface quality, electrical charges and the degree of stoichiometry of the material^23,24^. In the present work, the contributions of TDs themselves (with the exception of site #M2) to the inhomogeneity were relatively minor since the flat-type contact at site #F1, which did not involve a TD at the interface, was also well explained by the same model. One possible reason for the inhomogeneity of the barrier heights could be the electron-assisted deposition of Pt using an FIB system that was employed in this study, because the as-deposited Pt generated by this technique would be expected to include carbon-rich contaminants^26^. Although the Pt electrodes were effectively purified by the annealing process so as to obtain good rectifying characteristics for the Schottky contacts, low levels of carbon-based impurities likely remained at the interfaces even after annealing and might have resulted in spatial variations in contact quality.

### Reverse J-V-T characteristics of the flat-type contacts

**Figure 6:**
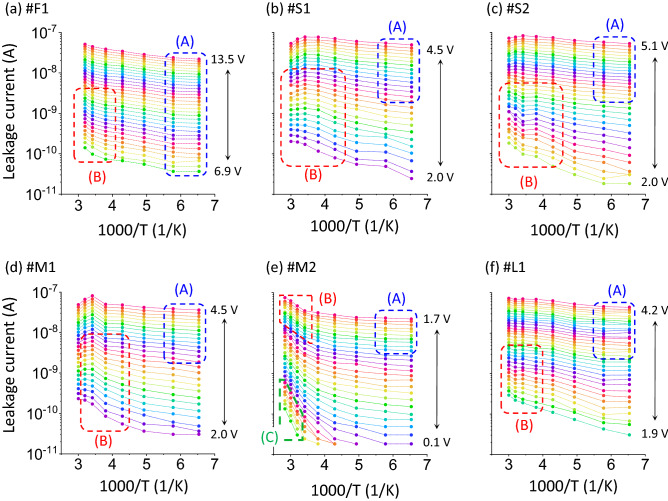
*I* vs. 1000/*T* data acquired under reverse bias conditions at different voltages for the (**a**) #F1, (**b**) #S1, (**c**) #S2, (**d**) #M1, (**e**) #M2 and (**f**) #L1 pits.

**Figure 7:**
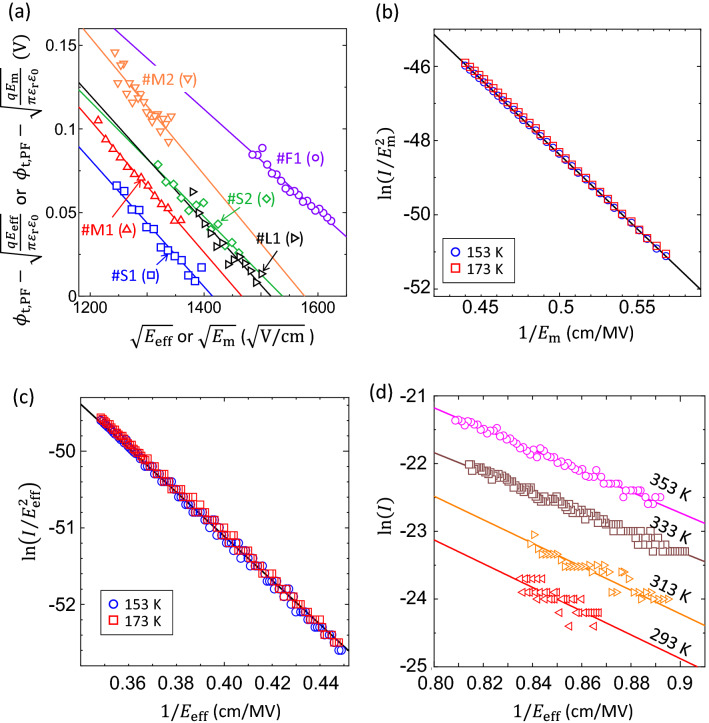
(**a**) ${\phi }_{\mathrm{t},\mathrm{PF}}-\sqrt{q{E}_{\mathrm{m}}/\pi {\varepsilon }_{\mathrm{r}}{\varepsilon }_{0}}$ vs. $\sqrt{{E}_{\mathrm{m}}}$ or ${\phi }_{\mathrm{t},\mathrm{PF}}-\sqrt{q{E}_{\mathrm{eff}}/{\pi \varepsilon }_{\mathrm{r}}{\varepsilon }_{0}}$ vs. $\sqrt{{E}_{\mathrm{eff}}}$ plots for data obtained in temperature range (B) for the #F1, #S1, #S2, #M1, #M2 and #L1 pits. (**b**) ln(*I*/${E}_{\mathrm{m}}$^2^) vs. 1/${E}_{\mathrm{m}}$ plots for the #F1 site and (c) ln(*I*/*E*_eff_^2^) vs. 1/*E*_eff_ plot for the #L1 pit. (**d**) ln(*I*) vs. 1/*E*_eff_ plots for data obtained in temperature region (C) for the #M2 pit. Solid lines in (a) and (d) represent the best fits to each data set while those in (**b**) and (**c**) are theoretical FNT curves calculated with *N*_D_ = 9.0 $\times$ 10^17^ cm^-3^, *T* = 153 K and the experimental barrier height.

Figure 6a–f provide plots of *I* vs. 1000/*T* based on the *J-V-T* data acquired under reverse bias conditions shown in Fig. 4a–f. In this section, the leakage current for site #F1 is analyzed to elucidate the electron transport mechanisms. Within the low temperature region below 173 K (referred to herein as region (A)) in Fig. 6a, there is almost no effect of temperature whereas there is a strong temperature dependence at lower voltages in the high-temperature range (region (B)). In the high-temperature region (B) the leakage current mechanism was likely Poole–Frenkel (PF) emission, which can be expressed mathematically as

1 $$I\propto E\mathrm{exp}\left(-\frac{q\left({\phi }_{\mathrm{t},\mathrm{PF}}-\sqrt{qE/\pi {\varepsilon }_{\mathrm{r}}{\varepsilon }_{0}}\right)}{kT}\right),$$

where *q*, *k*, *E*, ${\phi }_{\mathrm{t},\mathrm{PF}}$, ${\varepsilon }_{\mathrm{r}}$ and ${\varepsilon }_{0}$ are elementary charge, Boltzmann constant, the electric field strength, the depth of a trap level from the bottom of the conduction band, the relative permittivity of GaN and the permittivity of a vacuum, respectively^27–31^. The value of *E* was calculated as the maximum electric field concentration, *E*_m_, at the Schottky interface, expressed as

2 $${E}_{\mathrm{m}}=\sqrt{\frac{2q{N}_{\mathrm{D}}}{{\varepsilon }_{\mathrm{r}}{\varepsilon }_{0}}\left({\psi }_{\mathrm{bi}}-V-\frac{kT}{q}\right)},$$

where ${\psi }_{\mathrm{bi}}$ is the built-in potential^22^. The experimental values of *N*_D_ = 1.66 $\times$ 10^18^ cm^-3^, ${\psi }_{\mathrm{bi}}$=1.53 V (obtained from the room temperature *C-V* data) and ${\varepsilon }_{\mathrm{r}}$=10.4 were used for the *E*_m_ calculation^32^. Figure 7a presents a plot of ${\phi }_{\mathrm{t},\mathrm{PF}}-\sqrt{q{E}_{\mathrm{m}}/{\pi \varepsilon }_{\mathrm{r}}{\varepsilon }_{0}}$ vs. $\sqrt{{E}_{\mathrm{m}}}$ based on data acquired within region (B) for #F1. Here, the ${\phi }_{\mathrm{t},\mathrm{PF}}-\sqrt{q{E}_{\mathrm{m}}/{\pi \varepsilon }_{\mathrm{r}}{\varepsilon }_{0}}$ values were derived from linear fits to ln(*I*) vs. 1/*kT* plots using values generated within the temperature range of 263 to 313 K in Fig. 6a. The ${\phi }_{\mathrm{t},\mathrm{PF}}-\sqrt{q{E}_{\mathrm{m}}/\pi {\varepsilon }_{\mathrm{r}}{\varepsilon }_{0}}$ vs. $\sqrt{{E}_{\mathrm{m}}}$ plot was highly linear and ${\varepsilon }_{\mathrm{r}}$=6.19 and ${\phi }_{\mathrm{t},\mathrm{PF}}$=0.54 V could be obtained by fitting a straight line to the data. Because the resulting value of ${\varepsilon }_{\mathrm{r}}$ was in good agreement with previously reported data^27–31^, it was concluded that the PF mechanism was responsible for the leakage current in region (B). The temperature range over which the PF mechanism was dominant and the calculated values of ${\varepsilon }_{\mathrm{r}}$ and ${\phi }_{\mathrm{t},\mathrm{PF}}$ are summarized in Table 2.

**Table 2:** The ${\varepsilon }_{\mathrm{r}}$, ${\phi }_{\mathrm{t},\mathrm{PF}}$ and ${\phi }_{\mathrm{t},\mathrm{TAT}}$ values calculated by fitting the PF and TAT plots and the temperature ranges over which the PF and TAT mechanisms predominantly determined the reverse leakage current.

Label	${\varepsilon }_{\mathrm{r}}$	${\phi }_{\mathrm{t},\mathrm{PF}}$(V)	${\phi }_{\mathrm{t},\mathrm{TAT}}$(V)	Temperature (K)
#F1	6.19	0.54	–	263–313
#S1	3.98	0.54	–	233–333
#S2	4.84	0.56	–	233–333
#M1	3.69	0.58	–	233–313
#M2	3.41	0.65	0.63–0.66	293–353
#L1	4.02	0.57	–	263–333

Within the low-temperature region (A), the number of thermally-excited electrons would be expected to decrease with decreasing temperature. As such, electron tunneling across the thinner barrier potential in response to the high electric fields at the interface would become the primary source for the leakage current. This leakage current can be understood on the basis of the Fowler-Nordheim tunneling (FNT) mechanism^27,33^, expressed as

3 $$I\propto {E}^{2}\mathrm{exp}\left(-\frac{4\sqrt{q{m}^{*}}{\phi }_{\mathrm{b}}^{3/2}}{3\mathrm{\hslash }E}\right),$$

where ${\phi }_{\mathrm{b}}$, $\mathrm{\hslash }$, m* are the barrier height, Dirac's constant and the electron effective mass for GaN, respectively^22^. Under the low-temperature region, the effective donor concentration in the substrate were highly expected to decrease^24,34^. As presented in Fig. 7b, when using a slightly low donor concentration of *N*_D_ = 9.0 $\times$ 10^17^ cm^-3^, *T* = 153 K and m* = 0.20*m*_0_ (*m*_0_ is the free electron mass)^32^ for a calculation of *E*_m_ values, a plot of ln(*I*/${E}_{\mathrm{m}}$ ^2^) vs. 1/${E}_{\mathrm{m}}$ for site #F1 over temperature range (A) showed an excellent fit to a theoretical FNT curve obtained with the experimental value of ${\overline{\phi }}_{\mathrm{b}0}$=1.24 V. Therefore, the FNT mechanism is very likely to have been responsible for the leakage current within region (A). The value of *N*_D_ = 9.0 $\times$ 10^17^ cm^-3^ is used as the effective donor concentration in the subsequent section when analyzing the reverse leakage currents of the pit-type contacts in the low-temperature region below 173 K.

### Reverse J-V-T characteristics for pit-type contacts

As shown in Fig. 6b–f, the temperature dependence of the leakage current properties generated by the pit-type contacts decreases in a lower temperature (indicated by region (A)) and increases in a higher temperature (region (B)) similarly to that for the flat-type contact #F1. Based on the result of the #F1, it was highly likely that the leakage current mechanisms in (A) and (B) regions for pits #S1, #S2, #M1, #M2 and #L1 were the FNT and PF mechanisms, respectively. However, elucidation of the leakage current mechanisms associated with the pit-type contacts requires determination of precise electric field strength since the electric fields were expected to be crowded at the pit apex. Here we employed two ways to obtain effective electric field *E*_eff_ : one is derived from the experimental data fitting to the FNT model and the other from the finite-element-method simulations using COMSOL. In the former way, since the FNT mechanism is likely dominant in region (A) and the respective values of ${\overline{\phi }}_{\mathrm{b}0}$ for all pit-type contacts are known (see Table 1), *E*_eff_ can be determined so that the low temperature current data are well fitted to the theoretical FNT line. Figure 7c presents the representative plot of ln(*I*/*E*_eff_^2^) vs. 1/*E*_eff_ for #L1 pit in region (A), in which *E*_eff_ was determined by using the values of 1.30–1.36 as voltage-dependent ratios of *E*_eff_ with respect to *E*_m_ for the flat-type contact. Similar results were found for the #S1, #S2, #M1 and #M2 pits, giving the ratios for these contacts of 1.25–1.29, 1.34–1.45, 1.18–1.23 and 1.34–1.37, respectively. On the other hand, in the latter way, the COMSOL simulation revealed that the ratios of *E*_eff_ at the pit apex with respect to *E*_m_ for the flat-type contact for each pit size were 1.18–1.53 for the S-, 1.19–1.55 for the M-, and 1.19–1.57 for the L-pit in the reverse voltage range of 0–5 V (see Supplementary information). Comparison of these values indicates a good agreement with each other and the validity of *E*_eff_ as the electric field strength at the pit apex. Thus, in the following, we use the *E*_eff_ values obtained by the experimental data fitting for analysis of the leakage currents for the pit-type contacts.

Figure 7a presents ${\phi }_{\mathrm{t},\mathrm{PF}}-\sqrt{q{E}_{\mathrm{eff}}/\pi {\varepsilon }_{\mathrm{r}}{\varepsilon }_{0}}$ vs $\sqrt{{E}_{\mathrm{eff}}}$ (that is plots based on the PF emission model) in the high-temperature region (B) for the pit-type contacts. The ${\phi }_{\mathrm{t},\mathrm{PF}}-\sqrt{q{E}_{\mathrm{eff}}/\pi {\varepsilon }_{\mathrm{r}}{\varepsilon }_{0}}$ values derived from linear fits to the experimental *I* vs. 1000/*T* plots based on data acquired in range (B) of Fig. 6b–f. All the plots show a high degree of linearity and the ${\varepsilon }_{r}$ values derived from these fits are in the range 3.41–4.84. Because these values were close to previously reported data, PF emission was evidently responsible for the leakage current seen in region (B) for the pit-type contacts^27–31^. The calculated ${\phi }_{\mathrm{t},\mathrm{PF}}$ for the #S1, #S2, #M1 and #L1 sites are in the range 0.54 to 0.58 V. These values are very close to that obtained for site #F1 (${\phi }_{\mathrm{t},\mathrm{PF}}$=0.54 V), and so the trap levels inducing the PF-related leakage current conduction associated with the #S1, #S2, #M1 and #L1 pits are likely not related to the TDs but to other types of defects such as point defects. This finding is consistent with the observation that these ${\phi }_{\mathrm{t},\mathrm{PF}}$ values are very close to trap levels that have often been reported to originate from isolated point defects in GaN crystals (ranging from 0.57 to 0.61 V) by deep level transient spectroscopy studies^35^, indicating that these screw and mixed TDs had minimal effects on the leakage current conduction. In contrast, the ${\phi }_{\mathrm{t},\mathrm{PF}}$ value was calculated to be 0.65 V for the leaky #M2 pit, meaning that this level is significantly deeper than those for the other contacts. Therefore, this trap level likely had a different origin. The ${\varepsilon }_{\mathrm{r}}$ and ${\phi }_{\mathrm{t},\mathrm{PF}}$ values obtained from the PF analyses for all the contacts are summarized in Table 2.

Solely in the case of pit #M2, a significant leakage current was detected within a relatively small reverse voltage region (C), as can be seen in Fig. 6e, while no detectable leakage current occurred at such a small voltage for the other pit-type contacts. The leakage current was affected by temperature but was less likely to have been generated via the PF mechanism because the value of ${\varepsilon }_{\mathrm{r}}$ obtained from ${\phi }_{\mathrm{t},\mathrm{PF}}-\sqrt{q{E}_{\mathrm{eff}}/{\pi \varepsilon }_{\mathrm{r}}{\varepsilon }_{0}}$ vs. $\sqrt{{E}_{\mathrm{eff}}}$ plots in region (C) was too small (${\varepsilon }_{\mathrm{r}}$=0.71). Several studies have shown that the TAT model can possibly explain reverse leakage currents in GaN-based Schottky diodes^4,36^. This model can be summarized mathematically as

4 $$I\propto \mathrm{exp}\left(-\frac{4\sqrt{q{m}^{*}}{\phi }_{\mathrm{t},\mathrm{TAT}}^{3/2}}{3\mathrm{\hslash }E}\right) ,$$

where ${\phi }_{\mathrm{t},\mathrm{TAT}}$ is the depth of the trap level relative to the bottom of the conduction band^4^. Figure 7d presents a plot of ln(*I*) vs. 1/*E*_eff_ for pit #M2. The data obtained over the range of 293–353 K are seen to be highly linear, indicating that TAT was the dominant leakage mechanism in region (C). The ${\phi }_{\mathrm{t},\mathrm{TAT}}$ values calculated from fits to each data set were in the range of 0.63 to 0.66 V and were thus very close to the ${\phi }_{\mathrm{t},\mathrm{PF}}$ value for pit #M2, as shown in Table 2. Therefore, the trap states inducing both TAT- and PF-related leakage currents could have originated from the same source.

Leakage current conduction in metal/GaN Schottky barrier diodes with relatively high densities of TDs was often explained by the variable range hopping (VRH) along TDs. When the VRH conduction dominates the leakage current, ln(*I*) vs (1/*T*)^1/4^ plots become linear and the characteristics temperature, *T*_0_, calculated from a slope of a linear fit is generally in the range 10^6^–10^9^ K^37^. For GaN-based Schottky barrier diodes reported in earlier studies^29,37–39^, the *T*_0_ values are calculated to be 9.8 $\times$ 10^6^ – 6.15 $\times$ 10^9^ K. In our case, although ln(*I*) vs (1/*T*)^1/4^ plots of the *J-V-T* data for the pit-type contacts (#S1, #S2, #M1, #M2 and #L1) had good linearity within *T* = 173–263 K, calculated *T*_0_ values ranging from 3.4 × 10^4^ to 2.6 × 10^6^ K are relatively small compared to the reported ones. Therefore, it is unlikely that the VRH mechanism is dominant in the leakage currents for the pit-type contacts.

### MPPL observation

**Figure 8:**
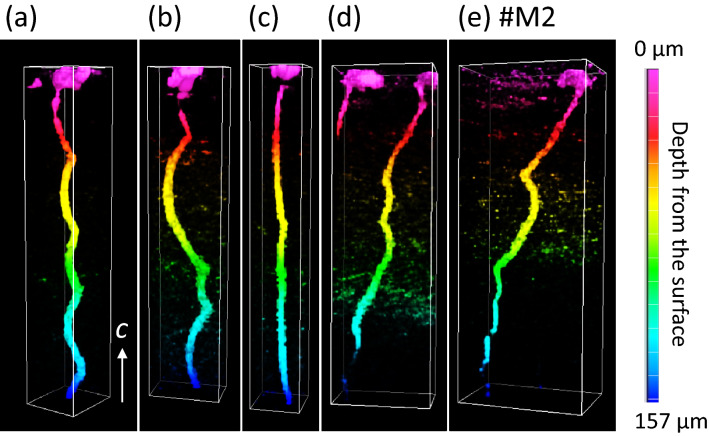
(**a**–**e**) Three-dimensional images of TDs under the M-pits. The TD in (**e**) is that under the #M2 pit. The gradient color of the TDs represents the depth from the surface.

Figure 8a–e show the three-dimensional morphologies of the TDs under the M-pits observed by MPPL. The majority of TDs under the M-pits, including #M2 (Fig. 8e), propagated with a helical morphology in contrast to linear propagation for edge and mixed TDs^20^. Although there were some variations in the detailed helical features such as amplitude, cycles of helices and meandering direction, no remarkable features specific to the TD for #M2 were identified. As a result, structural observations based on TEM and MPPL confirmed no significant differences in the Burgers vectors, the apparent core structures (in terms of open-core or closed-core) or propagation behavior between the screw TDs inducing the anomalously large leakage currents and those producing the small leakage currents. A recent study has demonstrated that all the **b** = 1***c*** closed-core screw TDs in GaN-on-GaN pn diodes coincided with the leakage spots^5^. In contrast, the present study revealed two types of **b** = 1***c*** closed-core screw TDs in the n-type GaN substrate: anomalous leaky TDs and less leaky TDs.

## Discussion

In previous work, first principles calculations showed that stable atomic core configurations and electronic structures of closed-core screw dislocations having **b** = 1***c*** were dependent on the conditions used to grow GaN crystals and that variations in these parameters would result in different leakage behavior for screw dislocations^19,40–43^. As an example, Nakano et al. reported that a stable atomic core structure of screw dislocations grown under a N-rich environment had no deep states in the bandgap, while a core structure formed under a Ga-rich condition created deep states^19,40^. Belabass et al. showed that screw dislocations having non-stoichiometric closed-core configurations with N atoms removed from the core had a metallic-like electronic structure that could provide highly conductive pathways in GaN crystals^41^. Based on these studies, the majority of the **b** = 1***c*** screw TDs in the present work had closed-core structures with no deep states inducing current leakage, while a very few** b** = 1***c*** screw TDs, such as that associated with pit #M2, had an atomically different closed-core structure with harmful deep states. These states resulted in significant reverse leakage currents in conjunction with the PF and TAT mechanisms. In the case of GaN-on-GaN pn diodes, it was reported that the condensation of magnesium impurities at the core of screw TDs could change the electronic structures of those TDs and produce leakage paths^19^.

The difference in the atomic core structures of screw TDs could be associated with a helical formation mechanism occurring during crystal growth. The helical structure is typically attributed to a nonconservative climbing motion of screw dislocations in which vacancies and/or interstitial atoms are adsorbed into the dislocation core so that the dislocation is deformed into a spiral shape^44^. According to this mechanism, the helical morphology and core structure are sensitive to fluctuations in the surrounding conditions, including concentrations of point defects (such as vacancies and impurities) and temperature^44,45^. Recently, it was shown that straight dislocations in GaN bulk crystals were deformed into a helical shape via segregation of vacancies in the dislocation core upon annealing at a high temperature of 1100 °C^46^. Therefore, the variation in the helical configuration of the TDs, as seen in Fig. 8a–e, implies that screw dislocations adsorbed point defects to configure the core structures in unique ways depending on the local growth conditions in the vicinity of each dislocation. On the basis of these considerations, it is predicted that the screw dislocation with the deep states inducing the large leakage current was subjected to atomic-scale modification of the dislocation core via climbing motion at the site where the growth environment was varied locally and significantly during the crystal growth. Several reports have provided experimental evidence of the conversion of screw dislocation core structures between open-core and closed-core in GaN crystals and the dependence of this transition on the growth conditions^11,47,48^. In particular, Hsu et al. found Ga droplets at the cores of screw TDs in GaN layers grown under Ga-rich conditions, and suggested that the preferential accumulation of Ga in screw dislocation cores generated a highly conductive core structure^13^. Therefore, we propose that the atomic-scale core reconfiguration of the closed-core screw TDs during the present growth process caused the abnormal leakage properties.

**Figure 9:**
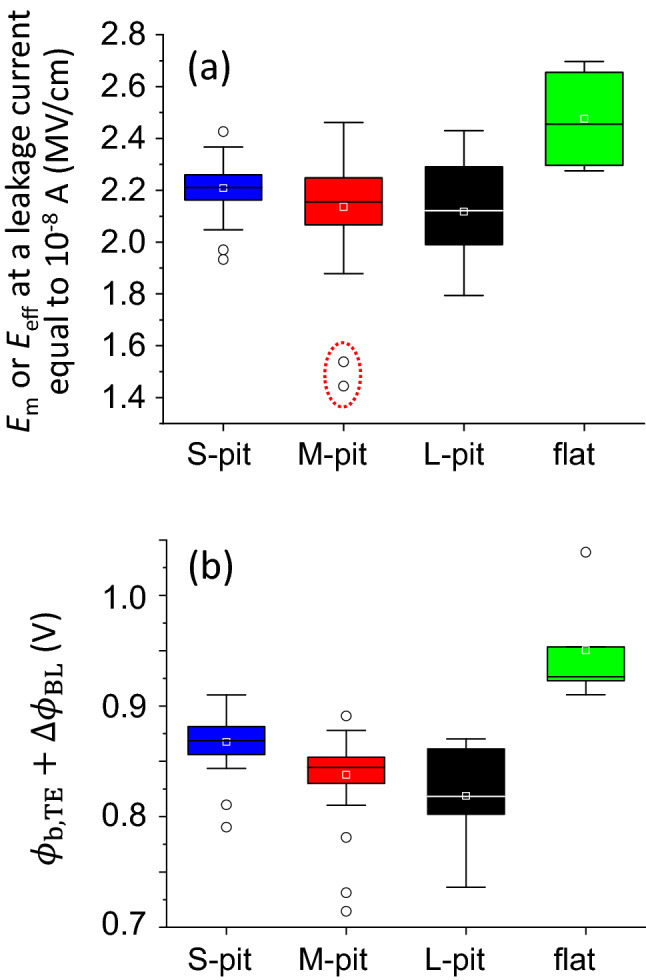
Box plots of (**a**) *E*_eff_ or *E*_m_ values at a leakage current of 10^–8^ A and (**b**) Schottky barrier heights. These results were obtained from the *J-V* data presented in Fig. 2a–d. In (**a**), two M-pits generating large leakage currents are indicated by a dotted circle.

Finally, we discuss effects of the majority of the TDs on the leakage current and barrier heights. Figure 9a shows box plots of *E*_eff_ and *E*_m_ for leakage currents of 10^–8^ A obtained using room temperature reverse *J-V* data for all the pit- and flat-type contacts in Fig. 2a–d. It is evident that *E*_eff_ was essentially unaffected by the pit size in the case of the pit-type contacts (except for two M-pits, at which large leakage currents were generated). The barrier heights for the pit- and the flat-type contacts were compared by taking account into the image-force lowering of the barrier height ($\Delta {\phi }_{\mathrm{BL}}$) in each case using *E*_eff_ and *E*_m_ at the zero-bias voltage, according to the equation^22^.

5 $$\Delta {\phi }_{\mathrm{BL}}=\sqrt{\frac{qE}{4\pi {\varepsilon }_{\mathrm{r}}{\varepsilon }_{0}}}.$$

Figure 9b shows a box plot of ${\phi }_{\mathrm{b},\mathrm{ TE}}$+ $\Delta {\phi }_{\mathrm{BL}}$ values for all the pit- and flat-type contacts. The difference in the average ${\phi }_{\mathrm{b},\mathrm{ TE}}$+ $\Delta {\phi }_{\mathrm{BL}}$ value for each pit size (0.868, 0.838 and 0.819 V for the S-, M- and L-pits, respectively) was at most approximately 0.05 V. Therefore, for most TDs having a ***c***-component, variations in the Burgers vector had only minor effects on the leakage current and the Schottky barrier height. Furthermore, the polar angle from the [0001] and azimuthal direction with respect to [11 $\overline{2 }$ 0] of the inclination of the TDs below the etch pits also exhibited minimal correlation with the leakage current (see Supplementary Figs. S3(a) and S3(b)). Therefore, the morphology of the screw and mixed TDs in the vicinity of the Schottky interface had little impact on the leakage currents.

In contrast, the leakage currents at the flat-type contacts were suppressed to a greater extent than those at the pit-type contacts, as can be seen in Fig. 9a. In addition, the average ${\phi }_{\mathrm{b},\mathrm{ TE}}$+ $\Delta {\phi }_{\mathrm{BL}}$ value of 0.95 V determined for the flat-type contacts was 0.11 V higher than the average for all the pit-type contacts (see Fig. 9b). Therefore, the smaller leakage currents generated by the flat-type contacts were likely to have been primarily associated with higher barrier heights than those produced by the pit-type contacts. Based on the similarity in the electrical conduction mechanisms for the pit-type contacts generating the small leakage currents (#S1, #S2, #M1 and #L1 pits) and site #F1, the difference in barrier heights between the pit- and flat-type contacts would not result from the intrinsic effects of the TDs but rather from extrinsic factors. In contrast to the Pt contacts on the ***c***-plane GaN surfaces at the flat-type contacts, in the case of the pit-type contacts, Pt was mainly deposited on semipolar inclined planes between the *c*- and *m*-planes that constituted the pyramidal etch pits. A recent study found that the Schottky barrier height for Ni/semipolar (10 $\overline{1 }$ 1) GaN contacts was higher than that for Ni/*m*-plane GaN contacts^49^. In addition, Yamada et al*.* reported that Schottky contacts on *c*-plane GaN had 0.16 V higher barriers than those on the *m*-plane^50^. Naganawa et al. also found a difference of 0.11 V in the barrier height between *m*- and *c*-plane contacts^51^. These reported values of barrier height differences are consistent with the value of approximately 0.11 V between the pit- and flat-type contacts obtained in the present study. Therefore, the lower barrier heights observed for the majority of pit-type contacts were likely due to the plane-orientation dependence of the Schottky barrier height. As a result, the effects of the TDs on the barrier height and the leakage current are thought to have been very small for the majority of the screw TDs with **b** = 1***c*** and the mixed TDs with **b** = 1***a*** + 1***c*** or **b** = 1*** m*** + 1***c***.

## Conclusion

The present study performed electrical and structural analyses of TDs with the screw-component (**b** = 1***a*** + 1***c***, 1***c*** or 1*** m*** + 1***c***). The results showed that a small number of the screw TDs generated a significant reverse leakage current by unique electrical conduction mechanisms associated with trap states that were distinctively deeper than those for other less leaky TDs. TEM and MPPL analyses of the TDs demonstrated that both the screw TDs generating the significant leakage current and those producing the small leakage current had the same Burgers vector (**b** = 1***c***), along with a similar closed-core structure and helical propagation morphology. These results strongly suggest that the leaky screw TDs had an atomically different closed-core configuration that could have been locally formed via interactions with point defects during the climbing process. In contrast, the majority of screw and mixed TDs generating small leakage currents had little impact on the current conduction. These data indicate that so-called killer defects in GaN-based power devices can be avoided by reducing the total density of screw TDs during the GaN bulk growth process. It is also important to precisely control the interactions between point defects and screw dislocations during device fabrication processes such as epitaxial growth and annealing procedures.

## Supplementary Information

Supplementary Information.

## Data Availability

The data that support the findings of this study are available from the corresponding author upon reasonable request.
