# Supplementary material for: Comprehensive analysis of current leakage at individual screw and mixed threading dislocations in freestanding GaN substrates
Source: Sci Rep. 2023 Feb 10;13:2436. doi: 10.1038/s41598-023-29458-3 (PMC9918472; doi:10.1038/s41598-023-29458-3)
Supplement: Supplementary file 1 — Supplementary Information. [file 41598_2023_29458_MOESM1_ESM.pdf]

SUPPLEMENTARY MATERIAL

Comprehensive analysis of current leakage at individual screw and mixed threading dislocations in freestanding GaN substrates

Takeaki Hamachi,[1,a)] Tetsuya Tohei,[1] Yusuke Hayashi,[1] Masayuki Imanishi,[2] Shigeyoshi Usami,[2] Yusuke Mori,[2] and Akira Sakai[1,b)]

[1] *Graduate School of Engineering Science, Osaka University, 1-3 Machikaneyama-cho, Toyonaka, Osaka 560-8531, Japan*

[2] *Graduate School of Engineering, Osaka University, 2-1 Yamadaoka, Suita, Osaka 565-0871, Japan*

LACBED analysis of the TD under the leaky M-pit

Figures S1(a) and S1(b) show LACBED images in which the dislocation under #M2 crosses specific Laue reflection lines and creates nodes at the intersections. According to the principle that **b** (defined as $[u, v, \overline{u+v}, w]$), $\boldsymbol{g}$ for a Laue reflection line crossing the dislocation, and the number of nodes (N) at the intersection satisfy the relationship $\boldsymbol{g}\cdot\mathbf{b}=N$,[1] Figures S1(a) and S1(b) were used to derive the equations,

$$
\begin{aligned}
&0u + 0v + 0(\overline{u+v}) + 8w = 8,\\
&-1u - 1v + 2(\overline{u+v}) - 6w = -6, \qquad \text{(S1)}\\
&0u + 1v - 1(\overline{u+v}) - 9w = -9.
\end{aligned}
$$

By solving Eq. (S1), the Burgers vector was determined to be **b**=0001=1$\boldsymbol{c}$.

Conventional thermionic emission model

The forward current density of the Schottky contact due to thermionic emission (TE) can be expressed as

$$J_{\mathrm{TE}} = J_{0,\mathrm{TE}}\left\{\exp\left(\frac{qV}{nkT}\right) - 1\right\} \tag{S2}$$

$$J_{0,\mathrm{TE}} = A^{*}T^{2}\exp\left(-\frac{q\phi_{\mathrm{b,TE}}}{kT}\right), \tag{S3}$$

where J_{TE}, $J_{0,\mathrm{TE}}$, n, $\phi_{\mathrm{b,TE}}$, T, A^*, q and k are the current density, saturation current density, ideality factor, barrier height, temperature, effective Richardson constant, elementary charge and Boltzmann constant, respectively.[2] Here, A^* is given by $4\pi qm^{*}k^{2}/h^{3}$, where m^* is the electron effective mass for GaN, and h is Planck's constant. Using $m^*=0.20m_0$ (m_0 is the free electron mass) gives a value of 24.0 $\mathrm{A/cm^2{\cdot}K^2}$.[3] The values of $\phi_{\mathrm{b,TE}}$ and n can be determined from a plot of ln(J) as a function of V in the linear region defined by $V > 3kT/q$. According to Eq. (S3), the values of $\phi_{\mathrm{b,TE}}$ and A^* can be determined by the activation energy plot (that is a conventional Richardson plot) based on a following equation,

$$\ln\left(\frac{J_{0,\mathrm{TE}}}{T^{2}}\right) = \ln(A^{*}) - \frac{q\phi_{\mathrm{b,TE}}}{kT}. \tag{S4}$$

Barrier inhomogeneity at the Schottky interface

In this model, the barrier height at the Schottky interface is assumed to have a Gaussian distribution, and the relationships between the experimentally measured barrier height, ϕ_{ap}, and the ideality factor, n_{ap}, and the temperature are given by

$$\phi_{\mathrm{ap}} = \bar{\phi}_{\mathrm{b0}} - \frac{q\sigma_{\mathrm{s}}^{2}}{2kT} \tag{S5}$$

$$\frac{1}{n_{\mathrm{ap}}} - 1 = -\rho_{2} + \frac{q\rho_{3}}{2kT}, \tag{S6}$$

where $\bar{\phi}_{\mathrm{b0}}$, σ_{s}, ρ_2 and ρ_3 are the zero-bias mean barrier height, zero-bias standard deviation of the barrier height distribution, voltage coefficient for the mean barrier height and voltage coefficient for the standard deviation, respectively.[4,5] A linear fit to a plot of ϕ_{ap} vs. $1/2kT$ allows σ_{s} and $\bar{\phi}_{\mathrm{b0}}$ to be determined from the slope and y intercept, respectively. A linear fit to a plot of $1/n_{\mathrm{ap}} - 1$ vs. $1/2kT$ also yields ρ_2 and ρ_3. Substituting ϕ_{ap} in Eq. (S5) for $\phi_{\mathrm{b,TE}}$ in Eq. (S4) gives a modified Richardson equation for the inhomogeneous barrier height model, written as

$$\ln\left(\frac{J_{0,\mathrm{TE}}}{T^2}\right)-\left(\frac{q^2\sigma_s^2}{2k^2T^2}\right)=\ln(A^*)-\frac{q\bar{\phi}_{\mathrm{b0}}}{kT}\ . \tag{S7}$$

The slope and y intercept of a linear fit to a plot of $\ln\left(J_{0,\mathrm{TE}}/T^2\right)-q^2\sigma_s^2/2k^2T^2$ vs. $1/kT$ give $\bar{\phi}_{\mathrm{b0}}$ and A^*, respectively.

Simulation of the electrical field distributions around pit-type contact Schottky interfaces

Establishing the electrical conduction mechanism associated with the pit-type contacts required an accurate assessment of the electric fields. The COMSOL Multiphysics software package was used to calculate the electric field distributions. Figure S2(a) presents a diagram of the pit-type contact model used for these simulations. A pyramidal etch pit as shown in Figs. 1(b) and 1(c) in the main text was simulated by performing a two-dimensional axial rotation of a right-trapezoid, such that the resulting pit shape was conical. A Pt/GaN Schottky contact was positioned along the slope of the etch pit plane and the *c*-plane surface region around the etch pit with a length of 1.9 µm. This length corresponded to the unintentional spreading-out of Pt during the electron-beam assisted deposition process. The backside surface was defined as the ohmic contact. The GaN thickness was set to 2.2 µm to reduce the computational resources required, and it was confirmed that the thickness had almost no effect on the calculation results so long as it was much greater than the width of the depletion layer at the interface. As shown in Fig. S2(b), scanning electron microscopy observations confirmed that the apex angle along the *m*-axis of the etch pit (defined as 2α) was dependent on the pit diameter (defined as $2r$). The average values of 2α and $2r$ for the S-, M- and L-pits were 105° and 1.9 µm, 96° and 2.6 µm, and 91° and 3.6 µm, respectively, and the electric fields of the etch pits were calculated using each average value. Observations using transmission electron microscopy (TEM) showed that the pit apexes had curvatures with radii, R_C, of 44-70 nm (Fig. S2(c)), and the R_C value of 44 nm was applied in the simulation. N_D=9.0×10^{17} cm^{-3} and T=153 K under which the FNT mechanism was the dominant factor affecting the reverse leakage current were also employed in the calculation. The work function of Pt, the electron affinity and the relative permittivity of GaN were set to 5.65 eV, 4.1 eV and 10.4, respectively.

Figures S2(d) provides the results of calculations of the electric field distributions at the Schottky interface around the M-pit apex. The inset presents the electric field distributions in the vicinity of the pit apex showing that the electric field was crowded at the pit apex with the curvature. Figure S2(e) shows the calculated maximum electric field strengths at the pit apex as functions of the applied reverse voltage for the S-, M- and L-pit-type contacts. The maximum electric fields were less dependent on the pit size. As an example, the maximum electric field strengths with a reverse voltage of 3 V were calculated as 2.30, 2.32 and 2.35 MV/cm for the S-, M- and L-pits. The maximum electric field strengths for the pit-type contacts were evidently larger than those for the flat-type contact due to the electric field crowding in Fig. S2(e). The ratios of the maximum electric field strength at the pit apex to the electric field strength for the flat-type contact were 1.18-1.53 for the S-, 1.19-1.55 for the M- and 1.19-1.57 for the L-pits in the range of 0-5 V.

Correlation between the inclination and the leakage current of the TDs

To assess the relationship between the leakage current and the inclination features of TDs, the polar angle from the [0001] and azimuthal direction with respect to $[11\bar{2}0]$ of the inclination of the TDs below the etch pits was analyzed by MPPL and the results were correlated with the E_{eff} values at a leakage current density of 1×10^{-3} A/cm^2. Note that the mixed TDs with **b**=1***a***+1***c*** and **b**=1***m***+1***c*** had linear propagation morphologies in contrast to the helical structure for the **b**=1***c*** screw TDs, as reported by our group.[6] Figures S3(a) and S3(b) provide the results, which indicate essentially no correlation between the inclination and the leakage current. Other morphological features, such as interactions with different dislocations, the spiral direction and the spiral cycle (especially in the case of the helical screw TDs) also exhibited minimal correlation with the leakage current.

REFERENCES

[1] Tanaka, M., Terauchi, M., & Kanayama, T. Identification of Lattice Defects by Convergent-Beam Electron Diffraction. *Microsc.* **40**, 211 (1991).

[2] Sze, S. M., & Ng, K. K. *Physics of Semiconductor Devices* (third edition) (Wiley, Hoboken, 2007).

[3] Barker, Jr., A. S. & Ilegems, M. Infrared lattice vibrations and free-electron dispersion in GaN. *Phys. Rev. B* **7**, 743 (1973).

[4] Yıldırım, N., Ejderha, K. & Turut, A. On temperature-dependent experimental *I*-*V* and *C*-*V* data of Ni/*n*-GaN Schottky contacts. *J. Appl. Phys*. **108**, 114506 (2010).

[5] Werner, J. H. & Güttler, H. H. Barrier inhomogeneities at Schottky contacts. *J. Appl. Phys*. **69**, 1522 (1991).

[6] Hamachi, T., Tohei, T., Hayashi, Y., Imanishi, M., Usami, S., Mori, Y., Ikarashi,. N. & Sakai, A., Propagation of threading dislocations and effects of Burgers vectors in HVPE-grown GaN bulk crystals on Na-flux-grown GaN substrates. *J. Appl. Phys*. **129**, 225701 (2021).

FIGURES and TABLES

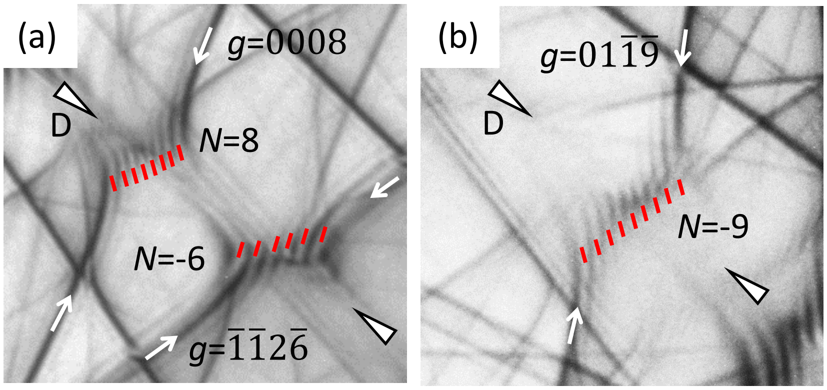

FIG. S1. (a), (b) LACBED patterns of TDs under the #M2 pit. The Laue reflection lines (indicated by white arrows) corresponding to a specific ***g*** split at the intersection with the dislocation line (indicated by arrowheads and the label D) to create nodes. The number of nodes is presented as *N*.

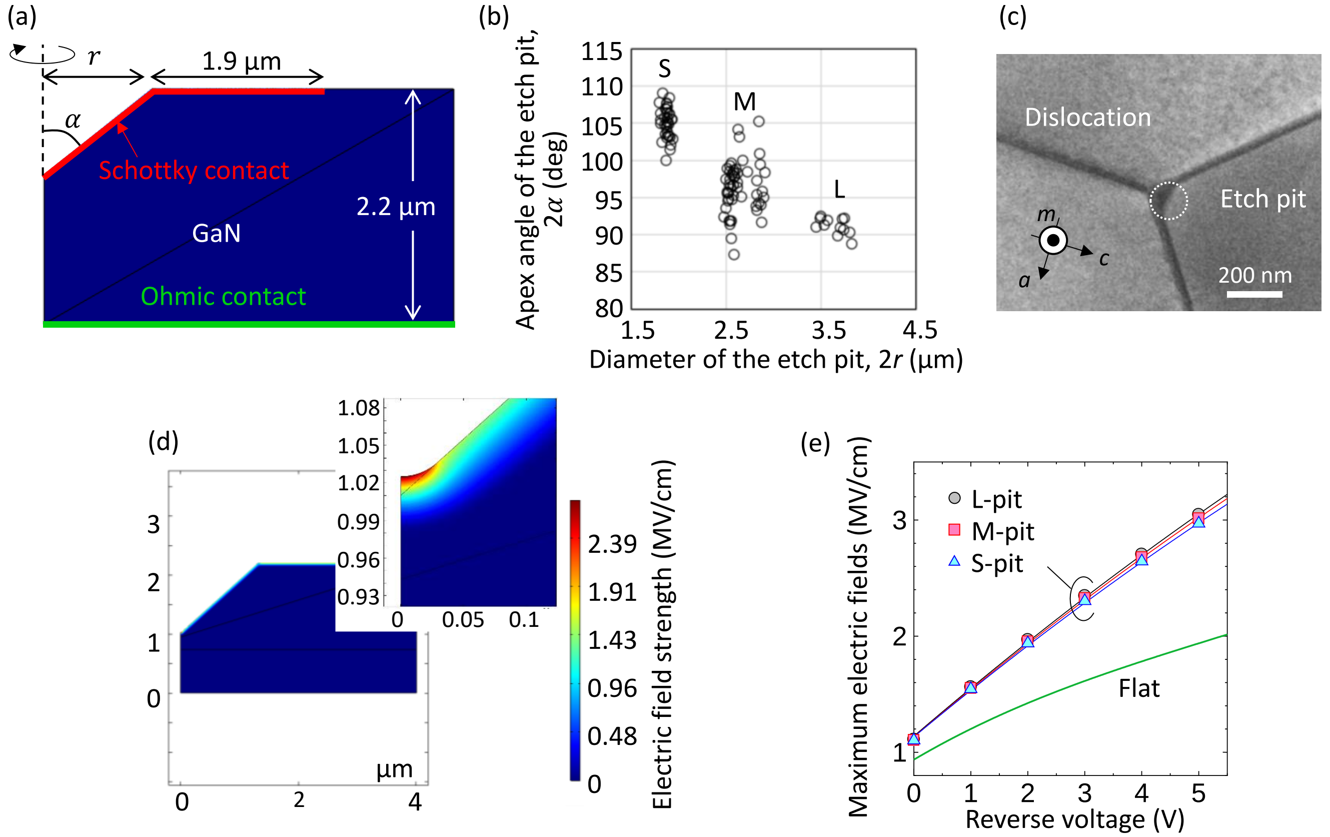

FIG. S2. (a) Diagram showing the pit-type contact model used for the simulations. (b) Relationship between the diameters (2*r*) and the apex angles along the *m*-axis (2*α*) of the etch pits. (c) A TEM image acquired in the vicinity of the pit apex, in which the curvature is indicated by the dotted circle. (d) Simulation results for the M-pit obtained using R_C=44 nm. The electric fields in the vicinity of the pit apex are magnified in the insets. (e) The maximum electric field at the pit apex for the pit-type contacts and at the interface for the flat-type contact as functions of the reverse voltage.

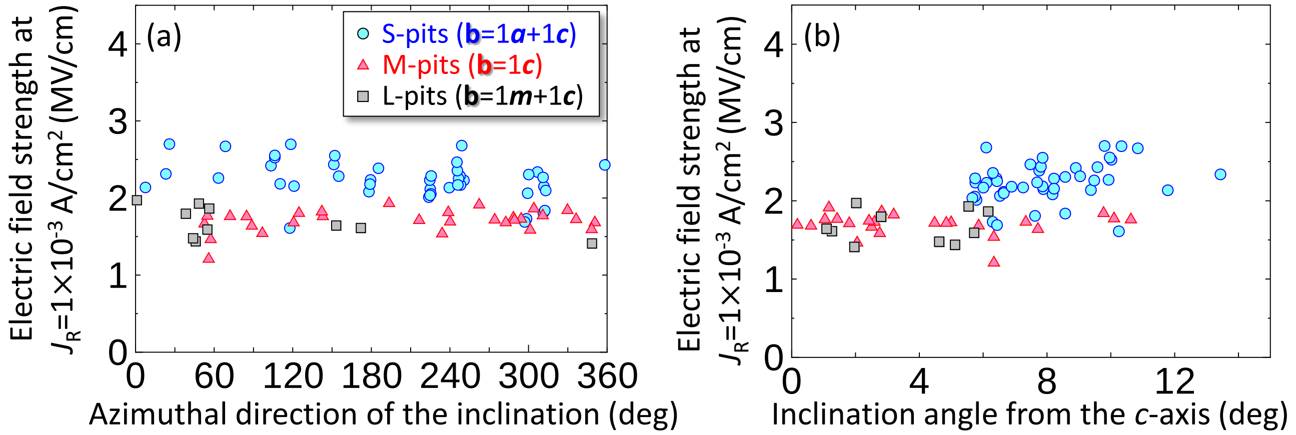

FIG. S3. E_{eff} values at a reverse leakage current density (J_{R}) of 10^{-3} A/cm^2 as a function of (a) the azimuthal direction with respect to $[11\bar{2}0]$ of the inclination and (b) the inclination angle from the [0001] of the TDs analyzed based on the MPPL data.
